# Investigation of Antibiotic Resistance of *E. coli* Associated with Farm Animal Feces with Participation of Citizen Scientists

**DOI:** 10.3390/microorganisms12112308

**Published:** 2024-11-13

**Authors:** Anna M. Timofeeva, Maria R. Galyamova, Dmitriy M. Krivosheev, Sergey Yu. Karabanov, Sergey E. Sedykh

**Affiliations:** 1 Institute of Chemical Biology and Fundamental Medicine SB RAS, 630090 Novosibirsk, Russia; anna.m.timofeeva@gmail.com; 2 Faculty of Natural Sciences, Novosibirsk State University, 630090 Novosibirsk, Russia; mgalyamova@gmail.com; 3 Chair of Biology and Chemistry, Vologda State University, 160000 Vologda, Russia; krivosheev@genotek.ru; 4 V.M. Gorbatov Federal Research Center for Food Systems, 109316 Moscow, Russia; karabans89@gmail.com

**Keywords:** citizen science, crowdsourcing, commensal bacteria, antibiotic resistance, antibiotic resistance genes, colistin, *mcr-1*, *vanA*, *vanB*, vancomycin, ampicillin, tetracycline, chloramphenicol, cefotaxime, ciprofloxacin

## Abstract

This paper presents the findings of a large-scale study on antibiotic resistance in bacteria found in farm animal feces across Russia. The study included 6578 samples of farm animal manure from 13 regions in Russia, with the help of citizen scientists. Molecular and microbiological methods were used to analyze 1111 samples of *E. coli*. The microbiological analysis focused on culturing the microorganisms present in the fecal samples on selective media for *E. coli* and evaluating the sensitivity of the bacteria to different antibiotics, including ampicillin, tetracycline, chloramphenicol, cefotaxime, and ciprofloxacin. The molecular analysis involved isolating the genomic DNA of the bacteria and conducting PCR assays to detect the *vanA*, *vanB*, and *mcr-1* antibiotic resistance genes. The results demonstrated significant differences in antibiotic sensitivity of the samples that are morphologically identical to *E. coli* from different regions. For example, 98.0% and 82.5% of *E. coli* and other fecal bacterial isolates from the Omsk and Vologda regions lacked antibiotic resistance genes, while 97.7% of samples from the Voronezh region possessed three resistance genes simultaneously. The phenotypic antibiotic sensitivity test also revealed regional differences. For instance, 98.1% of fecal bacterial samples from cattle in the Udmurt Republic were sensitive to all five antibiotics tested, whereas 92.8% of samples from the Voronezh region showed resistance to all five antibiotics. The high level of antibiotic resistance observed may be attributed to their use in farming practices. The distinctive feature of our research is that comprehensive geographical coverage was achieved by using a citizen science platform. Citizen scientists, specifically students from colleges and universities, were responsible for the collection and initial analysis of samples. The project attracted 3096 student participants, enabling the collection and analysis of a significant number of samples from various locations in Russia.

## 1. Introduction

Antibiotics have proven effective in both human and veterinary medicine, but the inappropriate use of these medications results in the emergence of antibiotic-resistant bacteria [1,2,3]. Livestock production is considered a major factor in the spread of antibiotic resistance genes in the environment. Antibiotic resistance genes have the potential to spread among animals, the environment, and humans through vertical gene transfer caused by the rapid growth of host bacteria or through horizontal gene transfer facilitated by mobile genetic elements [4]. The international community recognizes the gravity of the situation regarding the development of resistance to antibiotics, especially reserved antibiotics. It is possible for resistance genes to propagate on a global scale, including in regions that are already burdened with high levels of antimicrobial resistance. The emergence of multidrug-resistant bacteria can give rise to a grave public health concern [5].

The countries that have the highest proportion of global antibiotic consumption in livestock are China, the USA, Brazil, India, and Germany [6]. Most middle- and low-income countries lack a clear legislative framework for the use of antimicrobials in livestock [7]. The use of antibiotics in Russian agriculture is poorly regulated. Unfortunately, agricultural producers are not held accountable for the uncontrolled use of antibiotics. There is no system for assessing the need to use antibiotics in either livestock or crop production, and there is no control over the decisions of veterinarians and agronomists [8]. In total, several thousand different natural and synthetic substances are known to be used as antibiotics, while only a few types of antibiotics are monitored in Russia [9]. The All-Russian State Center for Quality and Standardization of Animal Drugs and Feed (VGNKI) often finds antibiotics in feed for farm animals, although this is prohibited. Research into the prevalence of antibiotic-resistant microorganisms in Russia is practically non-existent.

Several studies conducted in different countries described antimicrobials in livestock and reported resistance to ampicillin, tetracycline, and streptomycin [10]. The elevated level of resistance to tetracycline and ampicillin can be attributed to the extensive historical usage of these antimicrobials for therapeutic and prophylactic purposes [11]. A low percentage (<20%) of *E. coli* isolates resistant to at least one antimicrobial have been reported in cattle feces in countries such as the United States, Germany, and Denmark [12]. The widespread use of beta-lactams, aminoglycosides, fluoroquinolones, and macrolides has led to the antimicrobial resistance in Vietnam [13]. Resistance to at least one antibiotic was found in isolates of *E. coli* from 48% of calves in Sweden [14].

The two most important reserve antibiotics are colistin and vancomycin, and the detection of resistance to these antibiotics poses a serious public health threat. Colistin has been widely used in veterinary medicine in Asian, European, and North American countries, with its human use being limited due to its neuro- and nephrotoxicity [15,16,17]. However, the emergence of multidrug-resistant Gram-negative bacteria (such as Enterobacteriaceae, *Pseudomonas aeruginosa*, and *Acinetobacter baumannii*) has led to the increased use of colistin as an antibiotic of last resort [18]. The colistin resistance gene (*mcr-1*), which confers plasmid-mediated colistin resistance, was first discovered in China in 2015 and in Korea in 2016 [17,19]. Then, the *mcr-1* gene was identified in diverse bacterial species in over 50 countries [20]. Following the first discovery of the colistin resistance gene *mcr-1*, four additional *mcr* genes were described: *mcr-2* [21], *mcr-3* [22], *mcr-4* [23], and *mcr-5* [24]. The *mcr* genes are more commonly found in animal fecal isolates. However, numerous instances of human infection have been reported caused by strains carrying *mcr-1*, *mcr-3*, or *mcr-4* [25,26,27].

The resistance to vancomycin is associated with two types of gene clusters, designated *vanA* and *vanB* [28]. Vancomycin-resistant enterococci strains were first isolated in the late 1970s and have subsequently exhibited rapid worldwide dissemination [29]. The first documented clinical infection caused by vancomycin-resistant *S. aureus* was reported in Japan in May 1996 [30]. Later, vancomycin-resistant *S. aureus* strains were isolated in the United States, Australia, Europe, and other Asian countries [31].

Two main approaches are used to detect antibiotic resistance in farm animal fecal microorganisms: molecular (detection of antibiotic resistance genes based on PCR and sequencing) and microbiological (phenotypic detection of antibiotic resistance). Although culture-based methods are reference methods [32,33], they are time-consuming, more labor-intensive, and less cost-effective [34]. For example, the rapid automated GeneXpert vanA/vanB assay [33,35] provides results in less than an hour, while culture methods can take several days [36,37]. Another limitation of microbiological detection of vancomycin resistance genes is the difficulty in differentiating *vanA* and *vanB* genes: the *vanA* gene confers resistance to vancomycin and teicoplanin, whereas the *vanB* gene confers variable resistance to vancomycin, but susceptibility to teicoplanin [38]. In addition, according to CLSI guidelines, the disk diffusion method cannot reflect colistin resistance [39].

This study is distinguished by the active participation of citizen scientists in obtaining experimental results, with one objective of the study being to impart training in molecular biology and microbiology to students. Therefore, the crowdsourcing approach to determining antibiotic resistance in bacteria combined both approaches.

Despite the frequent use of antibiotics in veterinary medicine, there has been a lack of practical studies on the prevalence of antibiotic resistance in intestinal bacterial isolates from farm animals in Russia. In addition, the scope of research on microbial communities related to farm animals is usually restricted to the study of a single farm. The lack of large-scale studies on farm microbial communities in Russia can be attributed to the limited number of researchers, logistical challenges, and the high cost [40,41].

Overcoming the barriers discussed above is made possible by the involvement of citizen scientists. Citizen scientists team up to solve a scientific problem in communities guided by researchers [42]. It was our anticipation that using citizen science tools would enable us to gather a substantial number of samples and conduct their initial analysis, ultimately contributing to a comprehensive examination of the fecal microbiome of farm animals in Russia. In this study, the citizen science tools were used to collect 6578 samples of farm animal manure from 13 regions of Russia. Bacterial strains were isolated from animal fecal samples. Antibiotic resistance (ampicillin, tetracycline, chloramphenicol, cefotaxime, and ciprofloxacin) and prevalence of colistin (*mcr-1*) and vancomycin (*vanA* and *vanB*) resistance genes were analyzed for 1111 samples that were identified as *E. coli*. The research program involved the participation of 27 mentors and 3,096 college and university students. Involvement of citizen scientists became possible through the methods developed by scientists of Gorbatov Federal Research Center for Food Systems (Moscow, Russia), clear description of requirements for sample collection, metadata entry, and results analysis. Special kits with all the necessary reagents to conduct experiments in a crowdsourced format were developed. An educational program was developed to attract mentors to the project. This program provided training for mentors to conduct the collection of research material and its analysis with the students.

## 2. Materials and Methods

### 2.1. Citizen Science Recruitment and Mentor Training

This study was implemented in a network format, with scientific supervisors of the project (professional scientists) supervising the work of mentors and the mentors supervising the work of students. At the initial stage, “reference sites” were created based on educational institutions: colleges and universities having the necessary laboratory and educational infrastructure to participate in the research program. An arrangement was made to engage universities and colleges through invitation letters in the study. The outcome of this process led to the selection of 18 educational institutions situated across 13 regions in Russia. At each of the reference sites, a systematic approach was employed to arrange the collection, storage, accounting, analysis, and transportation of samples, followed by a meticulous review of metadata accuracy. One of the primary purposes of these sites was to involve students in conducting research. The study attracted a significant number of participants and mentors from agricultural and agricultural colleges, as access to agricultural facilities was a mandatory requirement.

In this study, crowdsourcing was employed for both sampling and experimentation purposes, specifically for isolating individual colonies and assessing their antibiotic resistance using the proposed methodologies. Specially designed kits containing all the requisite reagents for experiments were developed and distributed to the regions for research, alongside a comprehensive training program for students and their mentors.

The mentors were assigned the following tasks:

1. To organize and supervise the research team for sample collection and investigation;
2. To be familiar with the techniques of collecting, describing, storing, and transporting specimens according to research areas;
3. To organize the transfer of samples to V. M. Gorbatov Federal Research Center for Food Systems;
4. To organize the educational process based on the research activities of the program participants, to analyze the results with the participants, and to record the results under the requirements of educational institutions.

### 2.2. Sample Collection and Reagents

Special universal transportation kits were developed to collect manure samples. Two types of kits “kit for microbiological examination of samples” and “kit for examination of samples by PCR method” (MBU-Technology, Novosibirsk, Russia) were developed for examination of these samples. Each transport kit was provided with an individual unique number for sample labeling and instructions for use.

The protocol used in this work was developed specifically for this study. We relied on data from the study of antibiotic resistance of *E. coli* strains in feces of cattle [43,44], wild animals [45], and poultry [46,47].

The universal biological sample collection kit included the following:

1. Sample transportation medium;
2. Sample description questionnaire (species, age of the animal, life history of the animal, medication intake, farm coordinates, etc.);
3. Sample collection instructions.

The mentors organized field trips for the trainees to farms for sample collection and description. Fecal samples were collected from farm animals: cows, pigs, horses, and poultry (chickens, ducks, turkeys, and geese). The students, under the guidance of mentors, collected manure samples, filled out questionnaires about animal housing, and entered the data into a common electronic database. Bacterial strains corresponding morphologically and physiologically to *E. coli* were isolated from the fecal samples. The antibiotic resistance of *E. coli* was then analyzed using molecular and microbiological methods. Under the guidance of mentors, laboratory tests were conducted on the samples using one of the proposed methods.

### 2.3. Molecular Analysis of Samples

Genomic DNA was isolated from *E. coli* samples obtained by microbiological methods (see Section 2.4) by adding 100 µL of buffer containing 25 mM NaOH, 0.2 mM disodium EDTA, pH 12, incubated for 15 min at 95 °C, and centrifuged for 10 min at 12,000× *g* to the bacterial sample. The supernatant was neutralized by adding 200 μL of 40 mM Tris-HCl, pH 5.

Antibiotic resistance of bacteria was analyzed by PCR using the developed kit “kit for examination of samples by the PCR method” (MBU-Technology, Novosibirsk, Russia). The samples were analyzed for the presence of antibiotic resistance genes *vanA*, *vanB*, and *mcr-1*. The kit included instructions, pre-made reaction mixture containing one primer pair (*vanA*, *vanB*, or *mcr-1*), Taq polymerase, positive and negative controls, molecular weight markers, application buffer (1% SDS, 20 mM EDTA, pH 8.0, 30% glycerol and 0.005% bromophenol blue), DNA extraction solutions (cell lysis buffer: 25 mM NaOH, 0.2 mM disodium EDTA, pH 12, neutralization buffer: 40 mM Tris-HCl, pH 5), agarose, and TAE-50×. The primers used are summarized in Table 1.

The reaction mixture (14 μL) was supplemented with 1 μL of Taq polymerase and 5 μL of isolated DNA. Positive and negative controls were used for each reaction. The reaction was performed in an amplifier with the following parameters:

Stage 1: 95 °C for 15 min;

Stage 2: 35 cycles (95 °C for 10 s, 59 °C for 10 s, and 72 °C for 30 s).

The PCR products were separated in a 1.5% agarose gel, stained with ethidium bromide, and visualized using a UV transilluminator or a commercial Gel-doc system.

### 2.4. Microbiological Analysis of Samples

The “kit for microbiological examination of samples” (MBU-Technology, Novosibirsk, Russia) included instructions, endo-agar medium (Sredoff, St. Petersburg, Russia), nutrient agar—dry nutritional supplement based on fish meal hydrolysate (GRM) (Sredoff, St. Petersburg, Russia), Gram staining kit (Ekvus, St. Petersburg, Russia), antibiotic disks: ampicillin, ciprofloxacin, chloramphenicol, tetracycline, cephataxime (Bio-Rad, Hercules, CA, USA), test tube, and Petri dish with control strain *E. coli* ATCC922. Further antibiotic sensitivity analysis was performed for *E. coli* isolated from farm animal fecal samples.

The citizen scientists initially seeded the material in meat peptone broth, which was incubated for 18–20 h at 37 °C. Further analysis was performed by selecting colonies corresponding to the typical morphology of *E. coli*: cells are usually rod-shaped, about 2.0 μm long and 0.25–1.0 μm in diameter [48,49]. On agar, *E. coli* forms large, thick, grayish-white, moist, smooth, opaque or translucent colonies [50]. The bacteria were analyzed by Gram staining followed by microscopy. The isolates obtained were prepared for antibacterial susceptibility testing as follows: a daily pure culture of 2–3 colonies was added to 5 mL of PBS and mixed, and the final concentration was adjusted to 0.5 units according to the McFarland turbidity standard [51,52]. Next, the resulting suspension was applied to the surface of Petri dishes over the GRM medium using a sterile cotton swab. Then, the disks with antibacterial drugs were applied and incubated for 18–20 h at 37 °C. Similar manipulations were performed with the control strain *E. coli* ATCC 25922. The results were recorded by measuring the no-growth zone. First, the results of the control strain were evaluated, and if the growth inhibition zones fell within the specified ranges, the investigated isolates were counted. The results were interpreted according to CLSI criteria (M100 ED34 [53]), 2024 (https://clsi.org/standards/ 30 August 2024). The criteria included the presence or absence of the no-growth zone, which indicates the presence or absence of antibiotic resistance of the tested bacterial strain [54]. All subsequent studies examined only bacteria related to *E. coli*.

### 2.5. Analysis of Data Collected by Citizen Scientists

Under the guidance of mentors, volunteers entered the collected data into a joint database, and the data were finally analyzed in Microsoft Excel 2016. The data on antibiotic resistance of microorganisms associated with cattle and small farm animals for each region were analyzed. The qualitative data collected from the interviews were analyzed using a general inductive approach [55] to identify recurring and significant themes.

### 2.6. Statistical Analysis

The farm data (geographical location), farm animal data (animal type, housing conditions, sex, age, drug intake), and the analysis results were put into a Microsoft Excel spreadsheet. Next, descriptive statistics on animal housing and the prevalence of antimicrobial resistance or insensitivity were prepared. For each region and for each animal species, the resistance or insensitivity status of isolates to each antibiotic was determined.

Multivariate analysis was performed separately for each of the Russian regions for animal data and bacterial antimicrobial susceptibility testing results. The analysis was performed to identify the most important variables that explained variation in the dataset. The dataset comprised 38 data variables that were organized into 6 groups based on relatedness as follows: (1) farm animal species information; (2) herd information: farm location, breed distribution, herd size, pasture type, and animal housing conditions; (3) sampled animals’ life history and treatment history: method of fecal sample collection, date of fecal sample collection, whether the animal was treated with antimicrobials, and antimicrobials used for treatment; (4) analysis of antimicrobial resistance genes; and (4) antimicrobial susceptibility testing results. Variables within each group with loading weights ≥10 components were kept for interpretation. When necessary, the Mann–Whitney U-test with a significance level of 0.05 was used to test for statistical differences between independent groups. Qualitative data co-collected from the interviews were analyzed using a general inductive approach [56] to identify recurring and significant themes.

## 3. Results

### 3.1. Sample Collection and Analysis

**Figure 1:**
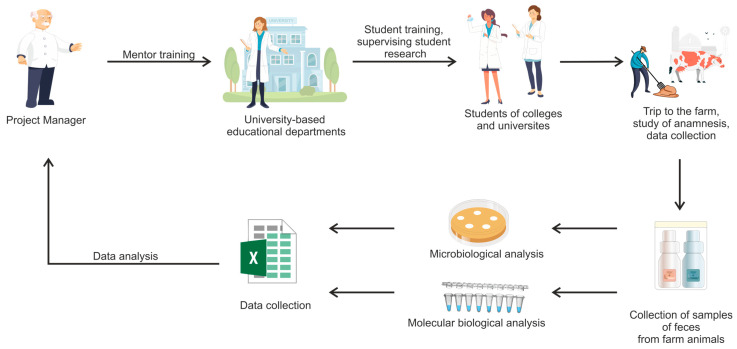
The general scheme of the study achieved with the use of citizen science tool. A project manager provides the general management of the project and training of mentors. Mentors train students and supervise the collection and analysis of samples. Sampling was conducted at the farm. Students and their mentors analyze the samples using one of the two proposed methods, and professional scientists compile the results in a table for further analysis.

The study was conducted using the citizen science tool. Figure 1 illustrates the general outline of the experiment. Initially, “reference sites” were organized in educational institutions: colleges and universities. The organization of sample collection, storage, accounting, analysis, and transportation was conducted at each reference site, with an additional step to verify the accuracy of the metadata completion. Under the supervision of mentors, students conducted the collection and analysis of fecal samples from farm animals. Two methods were used for the analysis of the samples: molecular and microbiological. The molecular analysis involved the isolation of genomic DNA from the bacteria being tested, followed by an examination for antibiotic resistance genes *vanA*, *vanB*, and *mcr-1*. The microbiological analysis involved antibiotic sensitivity testing by inoculating the bacterial culture in a “dense dash” onto the surface of a Petri dish and placing antibacterial disks on the surface of the medium. Experts (professional scientists) then collected the results in a single registry online for further analysis.

**Figure 2:**
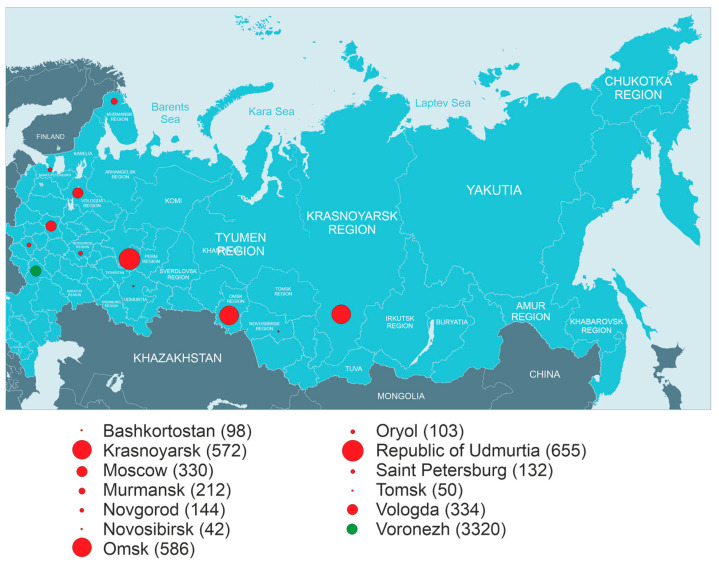
Regions of Russia covered by the study. The red circle indicates the region participating in the study, with the circle diameter proportional to the number of samples. The number of collected samples is indicated in brackets. The Voronezh region is indicated by a green circle.

The project started by analyzing the conditions of farm animal housing, collecting its anamnesis, and collecting manure samples for further research. In total, 6578 manure samples were collected from 13 regions of Russia (Figure 2).

The molecular and microbiological methods were used to analyze 1111 samples of *E. coli* from all regions of Russia (Table 2), with some regions showing preference for only one method of analysis (e.g., Moscow region, Omsk region, etc.). Further analysis was performed for the regions where over 10 samples were analyzed. Most of the samples analyzed are bovine fecal microorganisms (Table 3).

Most samples analyzed were cattle feces (881 samples, 79.3% of all samples). Analysis was conducted on fecal samples of pigs (113 samples, 10.2%), poultry (43 samples, 3.9%), horses (34 samples, 3.1%), and others. Some animals were fed vitamin and mineral supplements, and some were treated with antibiotic therapy. Below, we report only on those animals for which the fecal microbiome was analyzed (Table S1).

Among all animals, the cattle had the highest incidence of antibiotic use. A macrolide group antibiotic was administered to 55 cows in the Udmurt Republic, while a tetracycline antibiotic was given to 11 cows in the same region. The antibiotic drug “Tylosin” (active substance Tylosin) is renowned as the most popular one in the macrolide group. The most commonly used preparations for treating mastitis in cows include “Mastimax” and “Masticef”. These preparations are a combination of multiple antibiotics and contain active substances such as benzylpenicillin novocaine salt, dihydrostreptomycin sulfate, neomycin sulfate, dioxidine, dexamethasone sodium phosphate, gentamicin sulfate, and cephalexin.

The subsequent phase involved the analysis of collected samples of farm animal fecal microorganisms using molecular and microbiological methods, followed by a comparison of the data obtained with information regarding the antibiotics administered to farm animals. The following sections describe the results for those regions where at least 10 samples were analyzed: cattle from seven regions and pigs, poultry, and horses from the Voronezh region.

### 3.2. Molecular Analysis

**Figure 3:**
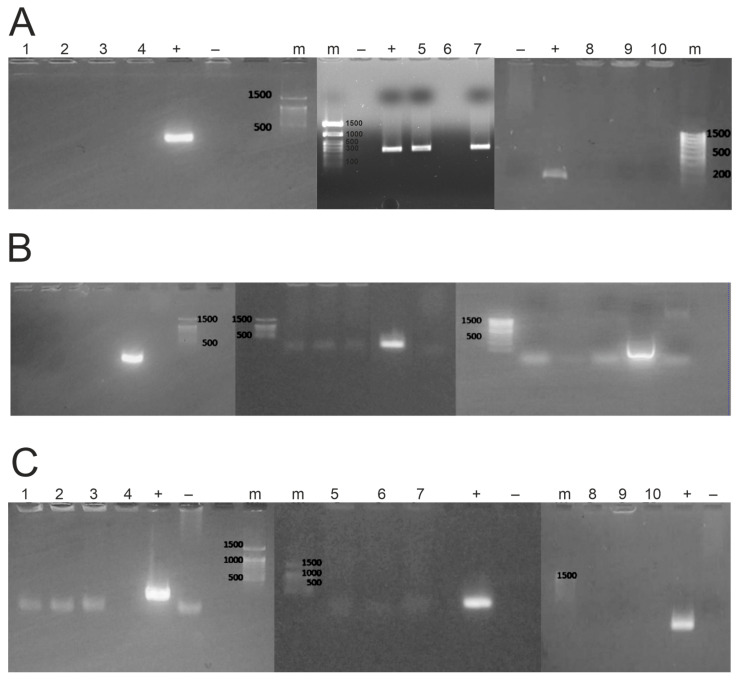
Screening of farm animal fecal microorganisms for antibiotic resistance genes: *vanA* (**A**), *vanB* (**B**), and *mcr-1* (**C**) by PCR. Presented here is an example of the analysis of 10 bacterial strains (1–10) derived from cattle fecal samples. The letter M is used to represent markers, while + and − are used to indicate positive and negative controls, respectively.

The microorganisms of farm animal feces were analyzed by PCR for resistance genes to three antibiotics: *vanA*, *vanB*, and *mcr-1*. Figure 3 shows an example of microbiological analysis for 10 bacterial strains. The length of the fragment corresponding to the *vanA* gene is 267 nucleotide pairs, the *vanB* gene is 237 nucleotide pairs, and the *mcr-1* gene is 253 nucleotide pairs.

**Figure 4:**
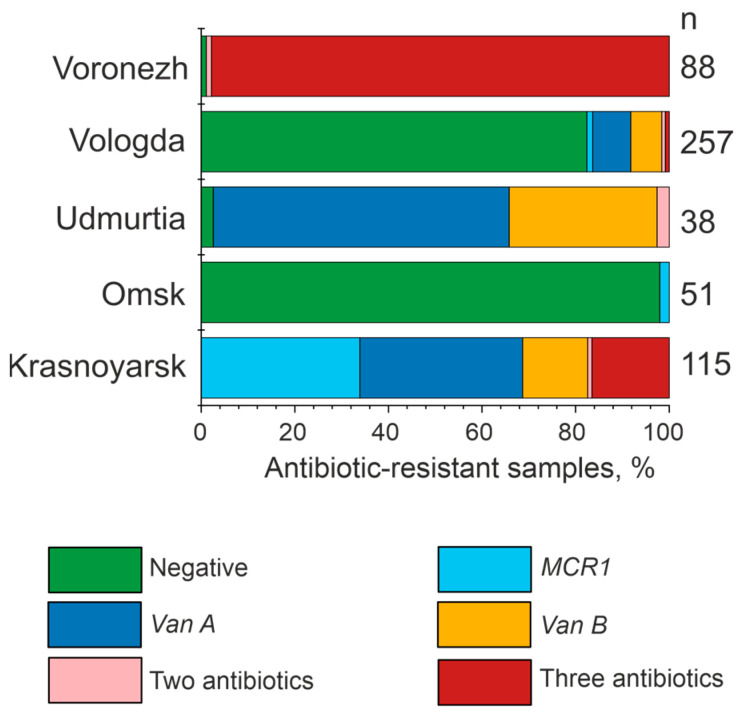
Analysis of antibiotic resistance of microorganisms associated with farm animals by PCR. A represents the data for cattle fecal microorganisms from five regions. n is the number of samples analyzed.

Tables S2 and S3 and Figure 4 contain a summary of all the results obtained, with significant regional differences. For example, in the Omsk and Vologda regions, most of the isolates under analysis lacked resistance genes to the antibiotics tested (98.0 and 82.5%, respectively), and in the Voronezh region, on the contrary, numerous samples resistant to three antibiotics simultaneously were detected (97.7% of samples).

This study has found a significant number of isolates carrying only the *mcr-1* gene (33.9% of all samples in the region) among 549 PCR-analyzed bovine fecal samples in the Krasnoyarsk region (Figure 4). Microbial resistance to colistin is a common phenomenon in farm animal fecal bacterial isolates [17,57,58,59] correlated with the use of colistin in veterinary medicine [57].

*E. coli* isolates containing *vanA* genes were identified in 8.2% of isolates in the Vologda region, 63.2% of isolates in the Udmurt Republic, and 34.8% in the Krasnoyarsk region. Similarly, *vanB* genes were found in 6.6% of isolates in the Vologda region, 31.6% of isolates in the Udmurt Republic, and 13.9% in the Krasnoyarsk region. The occurrence of isolates with both *vanA* and *vanB* genes detected simultaneously was rare, with only a few cases observed (2.6% in the Vologda region and 0.9% in the Krasnoyarsk region).

Some studies have reported the increase in the number of multidrug-resistant bacteria in recent years [60,61]. Of greatest concern is the Voronezh region, with 97.7% of cow fecal isolates found to be resistant to three antibiotics tested at the same time. A similarly high detection rate of resistance genes to three antibiotics was observed in the Krasnoyarsk region (16.5%). The high detectability of antibiotic resistance genes may be attributed to the widespread use of antibiotics in agricultural practice in these regions.

### 3.3. Microbiological Analysis

**Figure 5:**
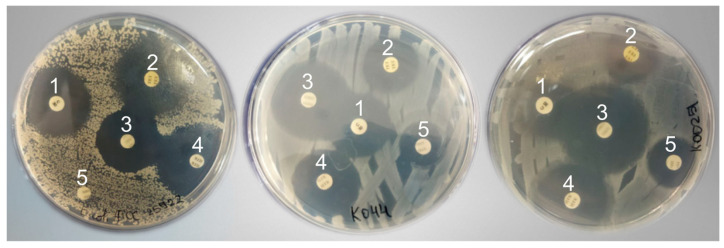
Example of microbiological analysis of a bacterial strain for resistance to five antibiotics: ampicillin (1), tetracycline (2), chloramphenicol (3), cefotaxime (4), and ciprofloxacin (5).The results of the analysis of bacterial samples from cattle feces are shown.

**Figure 6:**
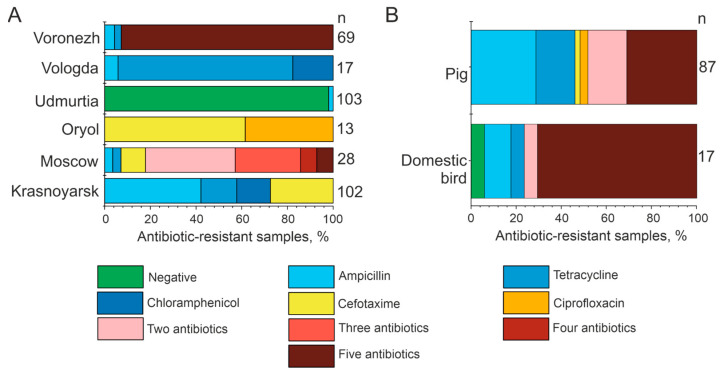
Analysis of antibiotic resistance of microorganisms associated with farm animals by microbiological methods. (**A**) The data for cattle fecal microorganisms from six regions of Russia, and (**B**) the data for different farm animals from the Voronezh region. n—the number of samples analyzed.

Microorganisms associated with farm animals were analyzed by microbiological methods for resistance to five antibiotics: ampicillin, tetracycline, chloramphenicol, cefotaxime, and ciprofloxacin (see Figure 5). Phenotypic antibiotic susceptibility testing in the present study revealed significant differences in the results depending on the region of study (Figure 6, Table S4). It was found that 98.1% of fecal bacterial samples of cows from the Udmurt Republic were susceptible to all five antibiotics analyzed. On the contrary, numerous microorganisms associated with cows from the Voronezh region were mostly resistant to five antibiotics simultaneously (92.8% of cow fecal bacteria samples). In the Vologda and Krasnoyarsk regions, 100% of microorganisms were found to be resistant to only one analyzed antibiotic. Within the Orel region, cattle fecal bacteria were resistant to cefotaxime (61.5% of samples) or ciprofloxacin (34.5% of samples). In the Moscow region, microorganisms showed resistance to both a single antibiotic and a combination of two to five antibiotics (Figure 6A).

Microorganisms resistant to ampicillin, tetracycline, cefotaxime, ciprofloxacin, and a combination of two or five antibiotics were found among bacteria in pig feces from the Voronezh region (Figure 6B). This study detected microorganisms with no antibiotic resistance, as well as those resistant to ampicillin, tetracycline, and a combination of two or five antibiotics among the bacteria of poultry feces from the Voronezh region. The most common were bacteria with multiple resistance (Figure 6B). Therefore, the Voronezh region of Russia is also categorized as the region demonstrating a significant prevalence of antibiotic-resistant microorganisms associated with poultry and pigs.

## 4. Discussion

Antibiotics are of great significance for maintaining farm animals, but their widespread use threatens the spread of antibiotic resistance. The prevalence of antibiotic-resistant bacteria and their antibiotic resistance genes is increasing due to the extensive use of antibiotics [62]. The most probable pathway for the transmission of antibiotic resistance involves the dissemination of antibiotic residues, antibiotic-resistant bacteria, and their genetic elements originating from the fecal matter of farm animals [63]. According to multiple studies, there is evidence suggesting that pastures [64], farm animal housing [65], land near farms, and field soils could contain higher concentrations of antibiotic residues, antibiotic-resistant bacteria, and antibiotic resistance genes [66,67,68].

It is a key objective of agricultural policy in numerous countries to control the spread and impact of antibiotic-resistant pathogens on humans and animals. For example, Germany has achieved a reduction in the prevalence of *mcr-1* from 8.1% in 2011 to 0.5% in 2014 in bacterial isolates of chickens [69]. The spread of antibiotic-resistant bacteria should be carefully monitored because of the high risk of pan-resistant pathogens in humans [70,71,72]. The use of citizen science-related tools allows researchers to organize the collection and initial characterization of specimens by unskilled citizen scientists rather than traveling to regions.

Here, we show that the resistance of *E. coli* isolates to antibiotics varied significantly depending on the region. For example, *E. coli* isolates with *vanA* genes were found in only 8.2% of isolates in the Vologda region, while *vanA* genes were found in 63.2% of isolates in the Udmurt Republic. The *mcr* genes associated with colistin resistance are widespread and have been described not only in cattle but also in pigs and poultry in different countries [73,74,75]. In Germany, *mcr-1* mediated colistin resistance in *E. coli* is detected predominantly in poultry chains, whereas the detection rate in cattle and pig isolates is much lower. These findings are in contrast to reports from Asian countries, where *mcr-1*-positive isolates are frequently identified in pig chains [17,76]. In China, a low prevalence of colistin-resistant *E. coli* was found in cattle (0.9%), but resistance among *E. coli* isolated from chickens and pigs was high (14% among chickens and 24% among pigs) [59].

The prevalence of enterococci resistance to vancomycin and other glycopeptide antibiotics has been extensively documented in numerous countries [77,78,79]. *VanA* is widely recognized as the prevailing resistance variant in numerous countries, excluding Australia and Sweden. However, most vancomycin-resistant enterococci cases have been linked to *vanB* [80,81,82,83]. The observation of *E. faecium* carrying both *vanA* and *vanB* was documented in 1993 in the UK [84], with such strains still rarely reported [85,86,87,88].

It is possible that swine production may play an important role in the spread of colistin resistance. For example, Enterobacteriaceae (mainly *E. coli*) isolates from pigs in Portugal tested positive for the *mcr-1* gene in 98% of cases [89]. In Ecuador, 41.9% of *E. coli* isolates from chickens and pigs were reported to contain *mcr-1* [90]. In Spain, the prevalence of colistin-resistant *E. coli* in pigs was 76.9% [91]. In China, a high prevalence of *E. coli* carrying *mcr-1* genes was also found in pigs (79.2%) [92]. In Europe, the prevalence of *mcr-1* genes in pigs ranged from 0.5 to 13.5% [93].

In the current study, three antibiotic resistance genes were detected simultaneously in 100% of cases (*vanA*, *vanB*, and *mcr-1*) in the analyzed fecal bacterial samples of pigs, horse, and poultry from the isolates obtained from the farms in Voronezh region. Hence, it can be concluded that the situation in the Voronezh region with antibiotic-resistant bacteria is extremely unfavorable, both on farms with cows and on farms of other types.

Analysis of microorganisms’ resistance to five antibiotics, i.e., ampicillin, tetracycline, chloramphenicol, cefotaxime and ciprofloxacin, also revealed significant differences depending on the region. The worst picture was observed in the Udmurt Republic and Voronezh region, where 98.1% and 92.8% of fecal bacterial samples from cattle, respectively, were resistant to all five analyzed antibiotics.

According to the European Medicines Agency [94], tetracyclines and penicillins (amoxicillin, ampicillin, and metampicillin) dominated the market as the most popular antibiotics used in livestock across 31 European countries from 2011 to 2018. Several countries, including Brazil, Argentina, Indonesia, Iran, Japan, Russia, Indonesia, Iran, Japan, and the USA, have formulated national strategies to address antimicrobial resistance. However, not all nations have successfully implemented measures to monitor the excessive use of antimicrobial agents [95].

Several countries have reported a high prevalence of tetracycline resistance among *E. coli* isolates from cattle feces [96,97]. The widespread use of tetracycline in human and veterinary medicine is attributed to its low cost, few side effects, and ability to stimulate animal growth [98,99,100,101].

Low rates (less than 2%) of resistance to fluoroquinolones (including ciprofloxacin) have been reported in Korea, Finland, Italy, and Sweden [102], with no cases reported in Canada, France, Denmark, Germany, and Australia [103]. Given that fluoroquinolones are of critical importance in human medicine, their use in veterinary medicine is reduced or banned in several countries [104]. Chloramphenicol is prohibited for animal feed in some countries, but it is still used in animal husbandry [105,106].

In Russia, these antibiotics continue to be actively used in agriculture. This may explain the high levels of resistance of bacteria associated with cattle feces to these antibiotics. The high proportion of strains with multidrug resistance may result from the widespread use of antimicrobial combinations in this region [107].

*E. coli* resistant to one or several antibiotics were found among bacteria in pig feces from the Voronezh region. In other countries, such as Korea [108,109,110], high levels of resistance to some antibiotics (ampicillin, streptomycin, and tetracycline) have also been reported in bacterial isolates from chickens and pigs. These results are consistent with tetracyclines, penicillin, phenicol (florfenicol), and aminoglycosides being the most marketed antibiotics for use in food animals in Korea [111]. An average of 29% of *E. coli* isolates collected from healthy pigs were found to be resistant to ampicillin in Denmark in 2012 [112].

In Voronezh, resistance to a combination of antibiotics was also observed in *E. coli* of poultry feces. This result is not unexpected. A high prevalence of *E. coli* isolates in broiler chickens showing resistance to ampicillin (87%), tetracycline (95%), ciprofloxacin (91%) and chloramphenicol (51%) has been reported in Brazil [113]. Resistance to tetracycline (93%) and chloramphenicol (50%) in poultry products from China has been found to be similar, while ampicillin resistance (99%) was higher, according to a study [114]. Similar findings have been documented in Mexico, where *E. coli* strains have exhibited significant resistance to ampicillin (92%), cefotaxime (78%), and tetracycline (75%) [115]. Therefore, the Voronezh region of Russia is also categorized as the region demonstrating a significant prevalence of antibiotic-resistant microorganisms associated with poultry.

In general, the high resistance to antimicrobial drugs is easy to explain because they are frequently used in farm animals, especially pigs and chickens [111]. Thus, the high level of resistance observed in chicken and pig isolates may reflect the use of these antimicrobials in poultry and pig farms.

The lack of correlation between documented antibiotic intake and resistant strains can be explained by resistant strains being developed during antimicrobial treatment and persisting for a long time in the intestinal tract of the animal after the treatment is discontinued [116].

This study was conducted with the help of citizen scientists. Under the guidance of professional scientists, citizen scientists unite to solve a multitude of scientific problems [42]. In recent times, several initiatives have been put into place to offer freshman students research opportunities [117,118]. The SEA-PHAGES Project, supported by the Howard Hughes Medical Institute and backed by the Phage Hunters Science Education Alliance, stands as the most successful discovery-oriented project in the United States [119,120,121,122]. Students participating in the SEA-PHAGES project learn about research methods, experiment design, and data interpretation. An outstanding feature of this project is its emphasis on enabling students to contribute to scientific discovery.

Citizen science is recognized as a highly effective tool for improving the productivity of screening processes. The availability of a comprehensive guide for non-professional researchers to conduct experiments enables the utilization of crowdsourcing not only for sampling but also for specific primary experiments [123]. Two different kits were designed specifically for conducting the required experiments in order to explore the antibiotic resistance of microorganisms found in farm animal feces.

In our project, the involvement of citizen scientists was based on networking, when the project supervisor interacts with mentors and trains them. Mentors interact with students and supervise their work. The participation of qualified mentors makes it possible to solve several significant problems of “scientific volunteering.” These are ensuring the safety of work, data verification, organizational difficulties of remote work with participants, change in generations of students conducting research, continuation of successfully conducted experiments of students, and others.

The purpose of involving citizen scientists in this project is to relieve scientists of the burden of conducting routine laboratory research. To ensure the reliability of the results obtained, the steps taken are as follows:

(1) We used simple, standardized methods for all participants.
(2) Positive and negative control samples were developed and submitted to the mentors. These controls provided for all of the methods indicated that the analysis was correct.
(3) We invited mentors: university and college teachers who worked with citizen scientists and monitored the quality of the work performed.
(4) Quality control and confirmation of the work performed by qualified scientists were carried out at every stage.

The involvement of citizen scientists has some limitations. Unfortunately, not all academic laboratories in colleges and universities are equipped to perform PCR; therefore, not all samples were analyzed. Citizen scientists performed the analysis that was available for their campus and laboratory. In addition, some participants collected more samples than they could analyze with the reagents provided.

Another limitation of this study is the uneven sampling across regions and across Russia. This problem can be addressed by selecting sample collection sites in advance when planning research involving citizen scientists. We understand all the limitations of this approach and see the merit of the study in the fact that student citizen scientists participated in an important study, learned microbiological and molecular methods, and conducted research that is important for scientists.

The project generated considerable enthusiasm, resulting in a significant number of participants and a significant number of samples collected and analyzed. The research results show that citizen scientists can effectively collect and analyze samples using only basic equipment. Citizen science is a promising field of scientific research. It allows citizen scientists to participate in scientific research and address important questions, such as the prevalence of antibiotic resistance in bacteria. Participation in citizen science projects like the current one can help motivate people to use antibiotics wisely in the long term.

## 5. Conclusions

This paper presents our findings on the regional variations in antibiotic resistance of *E. coli* associated with farm animals in Russia, highlighting the unique characteristics of each region. Unfortunately, the analysis conducted by citizen scientists in this study has primarily focused on bacteria from cattle feces, with a lack of comparable analysis on bacteria associated with other farm animals throughout Russia. A sufficient number of swine and poultry fecal samples were analyzed only in the Voronezh region. The examination of antibiotic-resistant bacterial isolates from farm animal guts is of great significance in the development of strategies aimed at mitigating antibiotic resistance in bacteria. Thus, monitoring antimicrobial resistance of bacterial isolates from animals on a regular basis may help to detect new antimicrobial resistance phenotypes in commensal and pathogenic bacteria in the food chain.

## Figures and Tables

**Table 1:** Primers used in the study.

Genes	Forward Primer	Reverse Primer
*mcr-1*	5′-CGGTCAGTCCGTTTGTTC-3′	5′-CTTGGTCGGTCTGTAGGG-3′
*vanA*	5′-GCCGGAAAAAGGCTCTGAA-3′	5′-TTTTTTGCCGTTTCCTGTATCC-3′
*vanB*	5′-GATTTGATTGTCGGCGAAGTG-3′	5′-TCCTGATGGATGCGGAAGA-3′

**Table 2:** Number of samples collected and analyzed across different regions of Russia.

Region	Samples Collected, n	Samples Analyzed by Molecular Methods, n	Samples Analyzed by Microbiological Methods, n	Total Number of Samples Analyzed, n
Bashkortostan	98	1		1
Krasnoyarsk region	572	115	102	217
Moscow region	330		28	28
Murmansk region	212			
Novgorod region	144			
Novosibirsk region	42	1		1
Omsk region	586	56		56
Oryol Region	103		13	13
Udmurt Republic	655	43	104	147
Saint Petersburg	132		1	1
Tomsk region	50			
Vologda Region	334	274	23	297
Voronezh region	3320	161	192	353

**Table 3:** Species diversity of farm animals that had their fecal samples analyzed.

**Molecular Analysis**								
**Region**	***Bos taurus*** **(cown), n**	***Sus domesticus*** **(pig), n**	Poultry *, n	***Equus caballus*** **(horse), n**	***Capra hircus*** **(goat), n**	***Ovis aries*** **(sheep), n**	**Other or Not Specified, n**	**Total, n**
Krasnoyarsk region	115		1					115
Omsk region	51			4		1		56
Udmurt Republic	38	2	4		1			43
Vologda Region	257	2	5	1	1	2	5	274
Voronezh region	88	22	13	20	9	3	6	161
Всегo	549	26	23	25	11	6	11	649
**Microbiological Analysis**								
**Region**	***Bos taurus* (cown), n**	***Sus domesticus*** **(pig), n**	Poultry *, n	***Equus caballus*** **(horse), n**	***Capra hircus*** **(goat), n**	***Ovis aries*** **(sheep), n**	**Other or Not Specified, n**	**Total, n**
Krasnoyarsk region	102							102
Moscow region	28							28
Oryol Region	13							13
Udmurt Republic	103		1					104
Vologda Region	17		2			3	1	23
Voronezh region	69	87	17	9	4	1	3	192
Всегo	332	87	20	9	4	4	4	462

* Poultry includes chicken, duck, turkey, and goose.

## Data Availability

The original contributions presented in the study are included in the article/supplementary material, further inquiries can be directed to the corresponding author.

## Supplementary Materials

The following supporting information can be downloaded at: https://www.mdpi.com/article/10.3390/microorganisms12112308/s1.
