# Supplementary material for: Investigation of Antibiotic Resistance of E. coli Associated with Farm Animal Feces with Participation of Citizen Scientists
Source: Microorganisms. 2024 Nov 13;12(11):2308. doi: 10.3390/microorganisms12112308 (PMC11596788; doi:10.3390/microorganisms12112308)
Supplement: Supplementary file 1 [file microorganisms-12-02308-s001.zip › microorganisms-3285949-supplementary.pdf]

microorganisms

MDPI

Supplementary Material

Table S1. Use of antibiotics for animals for which fecal microbiome analysis was performed.

Region	Type	Type of antibiotic	Number of animals
Omsk region	Cattle	macrolide antibiotic	1
Omsk region	Cattle	tetracycline antibiotic	1
Omsk region	Cattle	ß-lactam antibiotic	1
Omsk region	Cattle	combination drug	3
Udmurt Republic	Cattle	tetracycline antibiotic	11
Udmurt Republic	Cattle	macrolide antibiotic	55
Udmurt Republic	Poultry	macrolide antibiotic	1
Vologda Region	Cattle	combination drug	1
Voronezh region	Cattle	cephalosporin antibiotic	1
Voronezh region	Cattle	penicillin antibiotic	1

Table S2. Percentage of detection of antibiotic sensitivity of bovine microorganisms determined by PCR.

Region	Negative	MCR1	Van A	Van B	MCR1+Van A	Van A+Van B	MCR1+Van B	Van A +Van B +MCR1
Krasnoyarsk	0,0	33,9	34,8	13,9	0,0	0,9	0,0	16,5
Omsk	98,0	2,0	0,0	0,0	0,0	0,0	0,0	0,0
Udmurt Republic	2,6	0,0	63,2	31,6	0,0	2,6	0,0	0,0
Vologda	82,5	1,2	8,2	6,6	0,4	0,4	0,0	0,8
Voronezh	1,1	0,0	0,0	0,0	0,0	0,0	1,1	97,7

Table S3. Percentage of antibiotic susceptibility detection of microorganisms of different animal species determined by PCR in the Voronezh region.

Type of animal	Van A +Van B +MCR1
Horse	100
Pig	100
Poultry	100

Table S4. Percentage of antibiotic susceptibility detection of bovine microorganisms determined by microbiological methods.

	negative	ampicillin	tetracycline	chloramphenicol (levomycetin)	cefotaxime	ciprofloxacin	two	three	four	five
Krasnoyarsk	0,0	42,2	15,7	14,7	27,5	0,0	0,0	0,0	0,0	0,0
Moscow	0,0	3,6	3,6	0,0	10,7	0,0	39,3	28,6	7,1	7,1
Oryol	0,0	0,0	0,0	0,0	61,5	38,5	0,0	0,0	0,0	0,0
Udmurt Republic	98,1	1,9	0,0	0,0	0,0	0,0	0,0	0,0	0,0	0,0
Vologda	0,0	5,9	76,5	17,6	0,0	0,0	0,0	0,0	0,0	0,0
Voronezh	0,0	4,3	2,9	0,0	0,0	0,0	0,0	0,0	0,0	92,8

Table S5. Percentage of antibiotic susceptibility detection of microorganisms of different animal species determined by PCR in the Voronezh region.

	negative	ampicillin	tetracycline	cefotaxime	ciprofloxacin	two	five
Pig	0,0	28,7	17,2	2,3	3,4	17,2	31,0
Poultry	5,9	11,8	5,9	0,0	0,0	5,9	70,6
